# Differences between the white-tailed and mule deer chronic wasting disease agents after passage through sheep

**DOI:** 10.3389/fvets.2025.1632936

**Published:** 2025-07-22

**Authors:** Alexis J. Frese, Eric D. Cassmann, Jifeng Bian, Leisa Z. Mandell, Sura Smadi, M. Heather West Greenlee, Justin J. Greenlee

**Affiliations:** ^1^Virus and Prion Research Unit, National Animal Disease Center, Agricultural Research Service, United States Department of Agriculture, Ames, IA, United States; ^2^Department of Biomedical Sciences, College of Veterinary Medicine, Iowa State University, Ames, IA, United States; ^3^Oak Ridge Institute for Science and Education, Oak Ridge, TN, United States; ^4^Department of Veterinary Pathology, College of Veterinary Medicine, Iowa State University, Ames, IA, United States

**Keywords:** prions, chronic wasting disease, sheep, white-tailed deer, mule deer

## Abstract

**Background:**

Chronic wasting disease (CWD) is a fatal prion disease that affects the cervid species, including white-tailed deer (WTD) (*Odocoileus virginianus*) and mule deer (MD) (*Odocoileus hemionus*). Interspecies transmission of CWD is highly variable and dependent upon multiple factors. CWD of MD is transmissible to sheep after intracranial inoculation, with clinical signs and incubation periods similar to scrapie.

**Purpose:**

This study used sheep and transgenic mice to investigate the susceptibility of sheep to the CWD agent from WTD (WTD sheep CWD) when intracranially inoculated and to characterize the agent in subsequent passages.

**Methods:**

Fifteen Suffolk sheep with *PRNP* genotypes VRQ/ARQ, ARQ/ARQ, or ARQ/ARR were inoculated intracranially with the CWD agent from WTD. Western blots and enzyme immunoassays (EIA) were performed on brain and lymphoid tissues to analyze misfolded prion protein (PrP^Sc^) accumulation.

**Results:**

PrP^Sc^ was detected in 2 of 15 sheep (both ARQ/ARQ sheep) in the brainstem at the level of the obex, with a mean incubation period (MIP) of 39 months. In affected sheep, the distribution of PrP^Sc^ was limited to the central nervous system (CNS). Brain material from one positive sheep (ARQ/ARQ) was used to inoculate mice expressing the cervid (Tg12) and ovine (Tg338) prion protein gene. Passage of the WTD sheep CWD agent into cervidized mice resulted in an attack rate of 83% for PrP^Sc^ detection, with a mean incubation period of 377 days for all mice, while passage into ovinized mice resulted in no clinical signs or demonstration of PrP^Sc^. These results were compared to those of passage of MD CWD agent from sheep (MD sheep CWD) into cervidized and ovinized mice. There was an 86% attack rate in cervidized mice with a mean incubation period of 646 days for all mice and an attack rate of 100% in ovinized mice with a mean incubation period of 282 days.

**Conclusions:**

This data suggests that WTD CWD is unlikely to present a major risk to sheep but could be transmissible back to the cervid population. However, MD sheep CWD could present a risk to both the cervid and sheep populations.

## Introduction

Prion diseases or transmissible spongiform encephalopathies (TSEs) are the result of an accumulation of the misfolded prion protein (PrP^Sc^), ultimately leading to fatal neurodegeneration (1). CWD and scrapie are TSEs occurring naturally in the cervid and sheep host species (2). Scrapie was first reported in the United States in 1947 from an imported sheep (3), while CWD was first confirmed in 1980 from an MD in Colorado (4).

Sheep’s susceptibility to the scrapie agent can be determined by the host prion protein gene (*PRNP*). Codons associated with susceptibility include 136 V (valine), 154 R (arginine), and 171 Q (glutamine), a genotype of VRQ/VRQ. Codons associated with resistance to the classical scrapie agent include the genotype of ARR/ARR (5).

**Figure 1:**
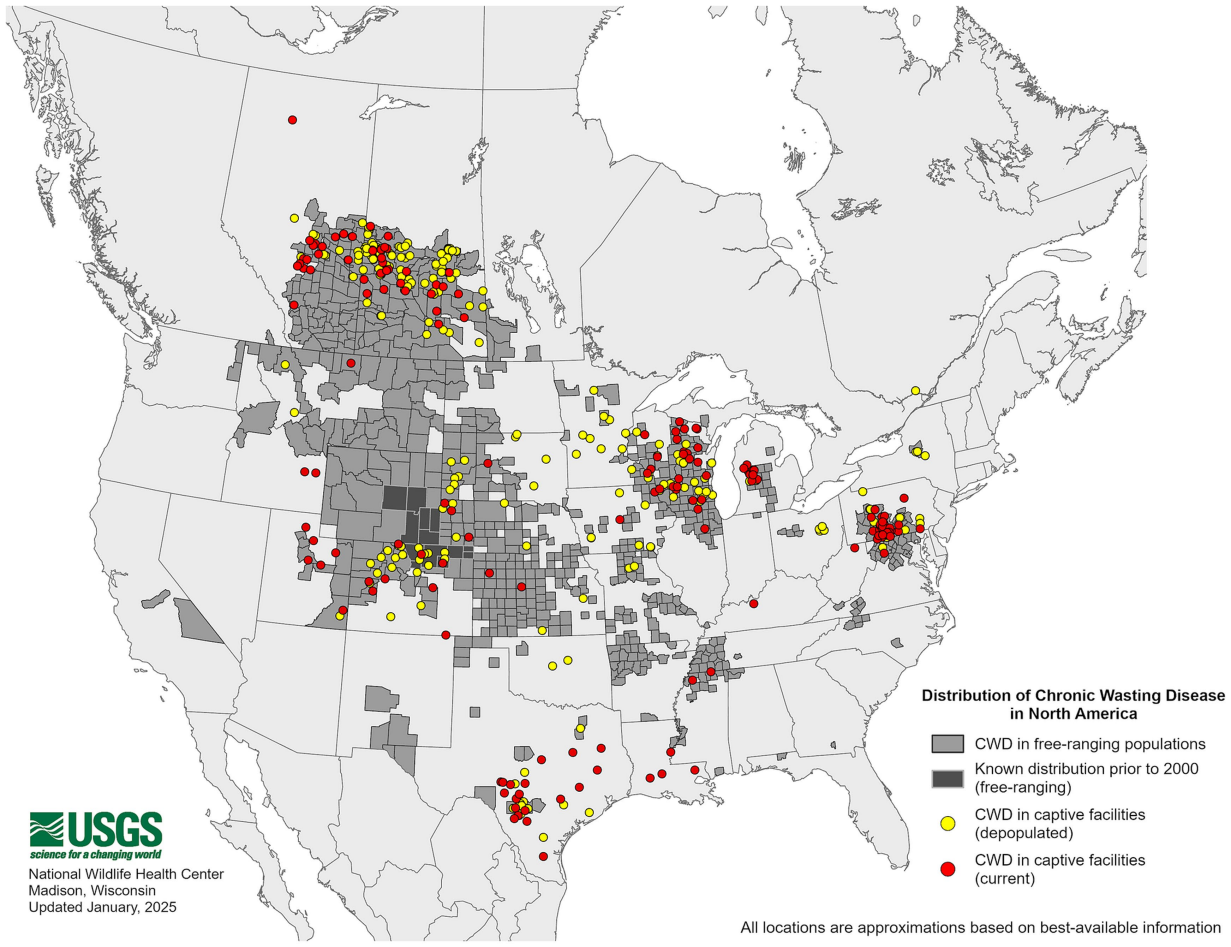
Distribution of chronic wasting disease in the United States. The map above shows free-ranging and captive cervids that tested positive for the chronic wasting disease agent. This map is courtesy of the United States Geological Survey (USGS).

**Figure 2:**
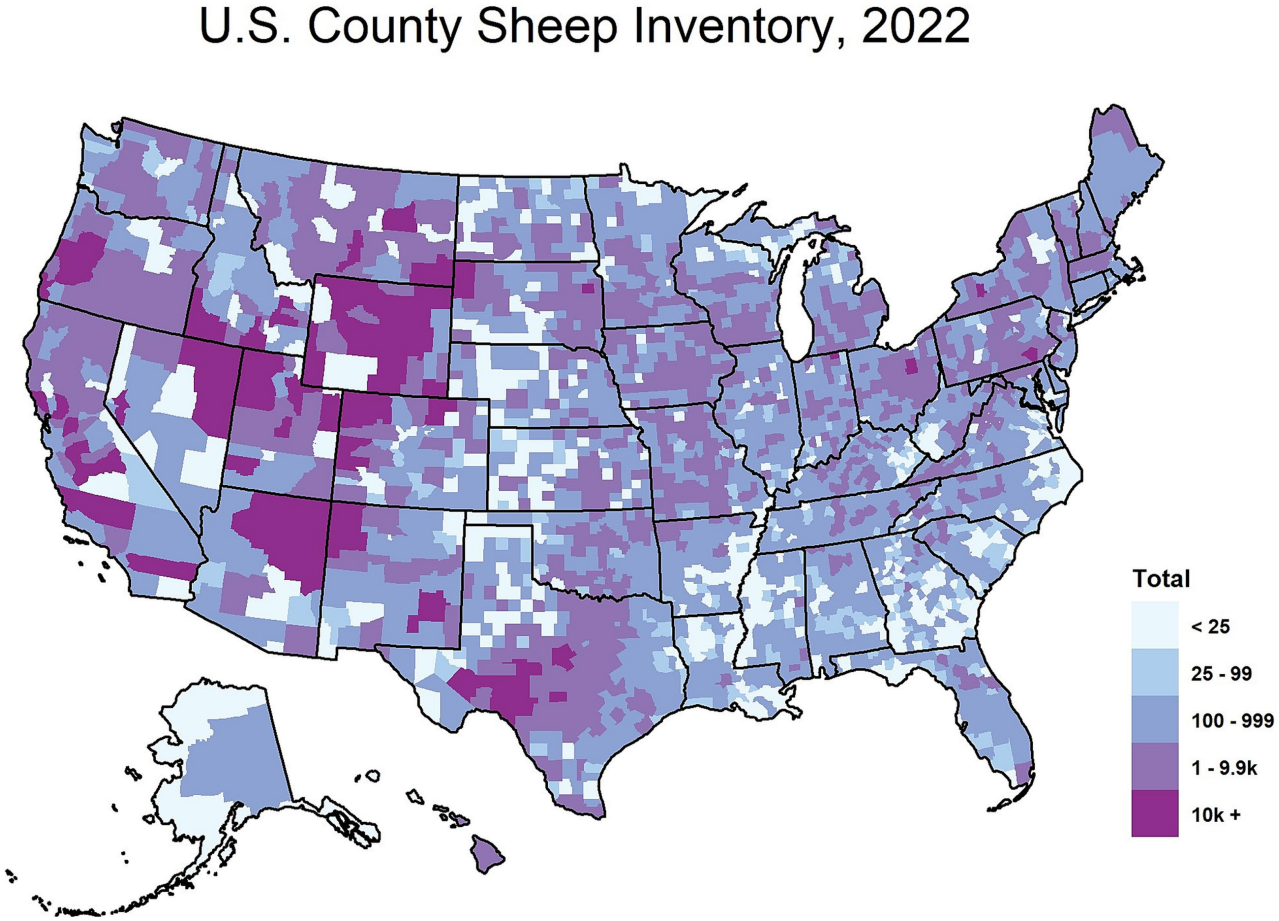
Total sheep inventory by county in the United States, 2022. The map above depicts the 2022 sheep inventory in the United States by county. This map is courtesy of the National Agricultural Statistics Service based on the 2022 United States Agricultural Census data.

**Figure 3:**
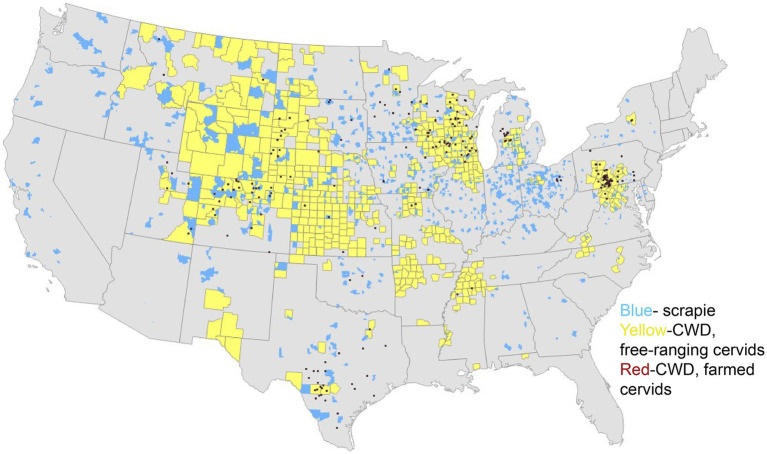
Distribution of scrapie and chronic wasting disease. The map above depicts scrapie events by zip code between 1988 and 2016 (blue) and positive cases of the chronic wasting disease agent in 2024, in free-ranging cervids (yellow) and captive cervids (red). This map is courtesy of Bryan Richards at the United States Geological Survey (USGS).

The origins of CWD are unclear, though one theory suggests that CWD could have originated from a cervid being exposed to the sheep scrapie agent (6–8). Both diseases exhibit the presence of PrP^Sc^ in the central nervous system (CNS) and lymphoid tissues (9, 10), raising the possibility of horizontal transmission between the two species (11). As of January 2025, 36 U.S. states have reported confirmed cases of CWD (Figure 1), with additional cases detected in countries such as Canada, Norway, Finland, Sweden, and South Korea (12). As CWD continues to become increasingly distributed among the cervid population, it is also increasingly contaminating the environment. Notably, the geographic distribution of CWD in both captive and free-ranging cervids overlaps with areas where sheep populations are present and scrapie has been detected (Figures 1–3). This overlap raises an important question of whether sheep could be assisting in the continued spread of CWD.

Previous work has shown that sheep are susceptible to both the WTD CWD agent (13) and the MD CWD agent (14, 15). This study demonstrates that sheep are oronasally susceptible to WTD CWD with a 14% attack rate, with PrP^Sc^ accumulating only in lymphoid tissues. Sheep are susceptible to the MD CWD agent intracranially with a 25% attack rate upon first passage and a 100% attack rate upon second passage. In the first passage of MD sheep CWD, PrP^Sc^ accumulation was present in both the CNS and lymphoid tissues of clinically affected sheep. Similarly, the second passage of MD sheep CWD also displayed PrP^Sc^ in the CNS and lymphoid tissues.

Oronasal inoculation most closely mimics natural routes of prion transmission; however, this study evaluates transmission following intracranial inoculation in sheep. Intracranial inoculation is commonly used in laboratory settings because it ensures efficient delivery of the agent to the central nervous system and bypasses barriers that may limit peripheral infection.

Building on previous findings, the current study focuses on CWD transmission involving WTD and MD, the two most widespread cervid species in the United States. These species are of significant focus due to their high population density in the United States and their potential role in the continual transmission of the CWD agent.

## Materials and methods

### Ethics statement

This study was carried out in accordance with the Guide for the Care and Use of Laboratory Animals (Institute of Laboratory Animal Resources, National Academy of Sciences, Washington, DC) and the Guide for the Care and Use of Agriculture Animals in Research and Teaching (Federation of Animal Science Societies, Champaign, IL). The protocol was approved by the Institutional Animal Care and Use Committee at the National Animal Disease Center (Protocol Number: 3985).

### Animal and inoculum procedures

A total of 15 four-month-old male lambs (Cheviot and Suffolk) of different *PRNP* genotypes (at codons 136, 154, 171) were obtained from a scrapie-free flock at the National Animal Disease Center (NADC) to carry out this study. The sheep were divided into three groups corresponding to different genotypes. Inoculations included six lambs with VRQ/ARQ genotypes, four with ARQ/ARQ genotypes, and five with ARQ/ARR genotypes. All sheep were inoculated with pooled CWD that was prepared from two WTD brains (genotypes 96GG) (16) combined with phosphate buffer saline (PBS) and gentamycin (100 μg/mL) to make a 10% w/v homogenate. To perform intracranial inoculations, the sheep were anesthetized with xylazine (up to 0.2 mg/kg) and ketamine (2 mg/kg) intravenously before 1 mL of inoculum was injected into their brain as previously described (14). All sheep were monitored daily for clinical signs of disease and euthanized with an intravenous overdose of pentobarbital sodium (85 mg/kg) once the disease presented or at the end of the study [55 months post-inoculation (MPI)].

Brain material from a CWD-positive sheep (418, Table 1) (ARQ/ARQ) intracranially inoculated with the CWD agent from WTD was used to intracranially inoculate mice expressing the cervid *PRNP* at approximately 2-fold (Tg12; 20 μL of 10% homogenate) or ovine VRQ *PRNP* at approximately 8-fold (Tg338; 20 μL of 1% homogenate) (17). Intracranial inoculations of mice were performed as previously described (18). Brain material from sheep positive for PrP^Sc^ after inoculation with the MD CWD agent was also inoculated into mice expressing the cervid *PRNP* (Tg12; 20 μL of 10% homogenate) and ovine VRQ/VRQ (Tg338; 20 μL of 1% homogenate) *PRNP*. All mice were monitored daily and euthanized when neurological clinical signs (ataxia, tremors, moribund, loss of body condition, or head tilt) were displayed or at the end of the study (approximately 700 days post-inoculation). All animals were confirmed positive or non-detectable by enzyme immunoassay (EIA) (IDEXX HerdChek BSE-Scrapie Antigen EIA test kit, Westbrook, ME), upon euthanasia. Sheep samples ran included the brainstem at the level of the obex, cerebrum, cerebellum, and retropharyngeal tonsils, while all mice brains were tested. All tissues collected were made as a 20% w/v homogenate.

**Table 1:** Sheep data.

Ear tag	Genotype	Incubation (MPI)	EIA results (OD)		
			Obex	Cerebellum	Cerebrum
403	AARRQQ	23	Neg	Neg	Neg
418	AARRQQ	27	3.9	Neg	Neg
424	AARRQQ	36	2.1	Neg	Neg
425	AARRQQ	31	Neg	Neg	Neg
408	AARRQR	35	Neg	Neg	Neg
439	AARRQR	55	Neg	Neg	Neg
441	AARRQR	36	Neg	Neg	Neg
432	AARRQR	55	Neg	Neg	Neg
433	AARRQR	28	Neg	Neg	Neg
415	AVRRQQ	48	Neg	Neg	Neg
423	AVRRQQ	47	Neg	Neg	Neg
404	AVRRQQ	25	Neg	Neg	Neg
410	AVRRQQ	55	Neg	Neg	Neg
412	AVRRQQ	31	Neg	Neg	Neg
413	AVRRQQ	51	Neg	Neg	Neg

MPI, months post-inoculation; OD, optical density; EIA, enzyme immunoassay.

### Western blot

Samples included in the western blots were all brainstem tissue at the level of the obex (sheep) and whole brain homogenates (transgenic mice). Tissues were homogenized to 20% (w/v) in phosphate buffer saline (PBS). First, a bicinchoninic acid assay (BCA) was performed on all samples to determine 50 μg of total protein. Tissue samples of 50–150 μL of were combined with 2.5 μL 20% sarkosyl/PBS, 2.5 μL proteinase K (PK) (1 mg/mL), and PBS to equal 25 μL. The samples were then incubated for 1 h at 37°C. Pefabloc was added to all samples to stop proteinase K (PK) digestion at room temperature for 20 min. Then, samples were combined with 2-ME and loading buffer and heated at 100°C for 5 min before gel electrophoresis.

Once the gel was transferred overnight, the membrane was blocked for 30 min with 3% BSA in 0.05% TBST. Signal detection was achieved using anti-PrP monoclonal antibodies, namely C-terminal binding SHA31, recognizing amino acids 145–152 on the human *PRNP* (Bertin Technologies, Montigny-le-bretonneux, France), and N-terminal binding 12B2, recognizing amino acids on the bovine *PRNP* at codons 101–105 (6) (Wageningen University and Research, The Netherlands), at a dilution of 1:10,000 as primary antibodies. The primary antibodies were incubated at room temperature for 1 h. The secondary antibody used was a biotinylated sheep anti-mouse IgG (1:400 dilution; GE Healthcare UK Limited, Amersham^™^, Buckinghamshire, United Kingdom) followed by streptavidin-HRP (1:10,000 dilution; GE Healthcare UK Limited, Amersham^™^, Buckinghamshire, United Kingdom). Both antibodies were incubated at room temperature for 1 h. ECL plus detection (1:10 dilution; Thermo Fisher Scientific, Carlsbad, CA) was used in conjunction with the iBright visualization system (Thermo Fisher, Invitrogen, Waltham, MA) for imaging.

### Enzyme immunoassay

Frozen tissues tested by EIA (IDEXX HerdChek BSE-Scrapie Antigen EIA test kit, Westbrook, ME) included the cerebrum, cerebellum, and brainstem at the level of the obex, palatine tonsil, and retropharyngeal lymph node. Tissues were homogenized to a 20% (w/v) concentration with PBS and then loaded onto a 96-well plate. The samples were run according to kit instructions, and a BioTek 800TS microplate reader was used to measure the optical density (OD) of each sample, differentiating positive and non-detectable samples. The negative cutoff value for mice samples was 0.18 plus the optical density of the negative control.

### Conformational stability assay

Brain homogenates (30 μg/μL) were denatured with three times the amount of guanidine hydrochloride (GdnHCl; 0–5.5 M) (Millipore-Sigma, G7294, Burlington, MA) for 1 h at room temperature. Utilizing a Bio-Dot Microfiltration apparatus (Bio-Rad, Hercules, CA), samples were filtered through Amersham Protran nitrocellulose membranes (Cytiva, Marlborough, MA) before washing twice with PBS. The membranes were air-dried for 1 h, then incubated at 37°C for 1 h with 5 μg/mL proteinase K (PK) in cell lysis buffer (50 mM Tris-HCl, pH 8.0, 150 mM NaCl, 0.5% sodium deoxycholate, and 0.5% Igepal CA-630). To inactivate the PK, 2 mM phenylmethylsulfonyl fluoride was put on the membranes, and 3 M guanidine thiocyanate in Tris-HCl (pH 7.8) was added for 10 min at room temperature. The membranes were washed with PBS four times, blocked with 5% non-fat milk in TBST for 1 hour, and probed with the primary antibody of SHA31 at a dilution of 1:5,000 (Bertin Technologies, Cat. A03213, France) overnight at 4°C. Primary antibody was followed by an HRP-conjugated goat anti-mouse IgG secondary antibody (1:5,000 dilution; Southern Biotech, Birmingham, AL). ECL Plus was used to develop membranes (1:10 dilution; Thermo Fisher, Scientific, Carlsbad, CA), and the iBright (Thermo Fisher, Invitrogen, Waltham, MA) was used for imaging. The AzureSpot Pro analysis software (Azure Biosystems) was used to analyze the signals of three biological replicates. The undenatured PrP^Sc^ (Fapp) was then plotted on the denaturation curve as a function of GdnHCl concentration using a non-linear least-squares four-parameter sigmoidal dose–response regression. The GraphPad Prism software (San Diego, CA) was used to calculate the half-maximal denaturation concentration, [GdnHCl]_1/2_, followed by a Student’s *t*-test to assess the statistical significance of [GdnHCl]_1/2_.

## Results

### Sheep results

For sheep intracranially inoculated with the WTD CWD agent, the mean incubation period was 39 months post-inoculation (MPI) for all sheep before death or euthanasia (Table 1). EIA was used to detect the amount of misfolded protein in CNS and lymphoid tissues after necropsy. No sheep showed unequivocal clinical signs of neurological disease; however, two sheep (418 and 424) tested positive for PrP^Sc^ by EIA on the brainstem at the level of the obex tissues (Table 1). All sheep displayed no detection of PrP^Sc^ in lymphoid tissues by EIA. All sheep cerebellum and cerebrum tissues were non-detectable when tested (Table 1).

### Sheep western blot

**Figure 4:**
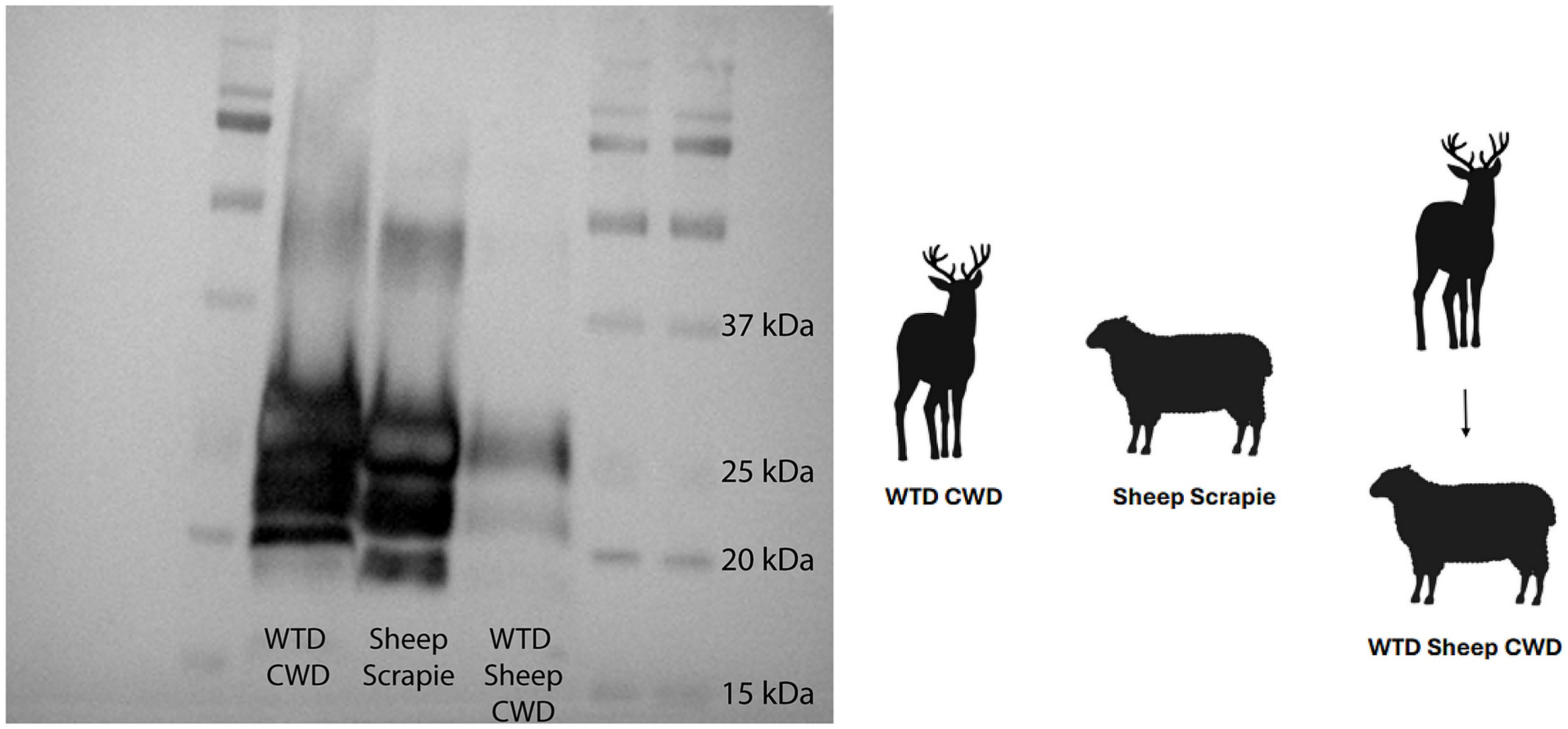
White-tailed deer sheep chronic wasting disease western blot. Western blot done on white-tailed deer with the chronic wasting disease agent, sheep with scrapie, and sheep with the white-tailed deer chronic wasting disease agent. All tissues on the blot are the brainstem at the level of the obex. Monoclonal antibody SHA31 was used to probe for PrP^Sc^.

In order to examine the molecular profile of brain tissue from WTD sheep CWD, a western blot was used to compare the brainstem at the level of the obex with brain samples from WTD inoculated with the CWD agent, and sheep inoculated with the scrapie agent. The results showed that the WTD sheep CWD agent (sheep 418) had a molecular profile similar to sheep scrapie, which was different from WTD with the CWD agent (Figure 4). The WTD CWD agent migrated a smaller distance, as the unglycosylated band is heavier at 20 kDa, compared to the WTD sheep CWD and sheep scrapie agent, which traveled a longer distance.

### Cervidized mice results

**Figure 5:**
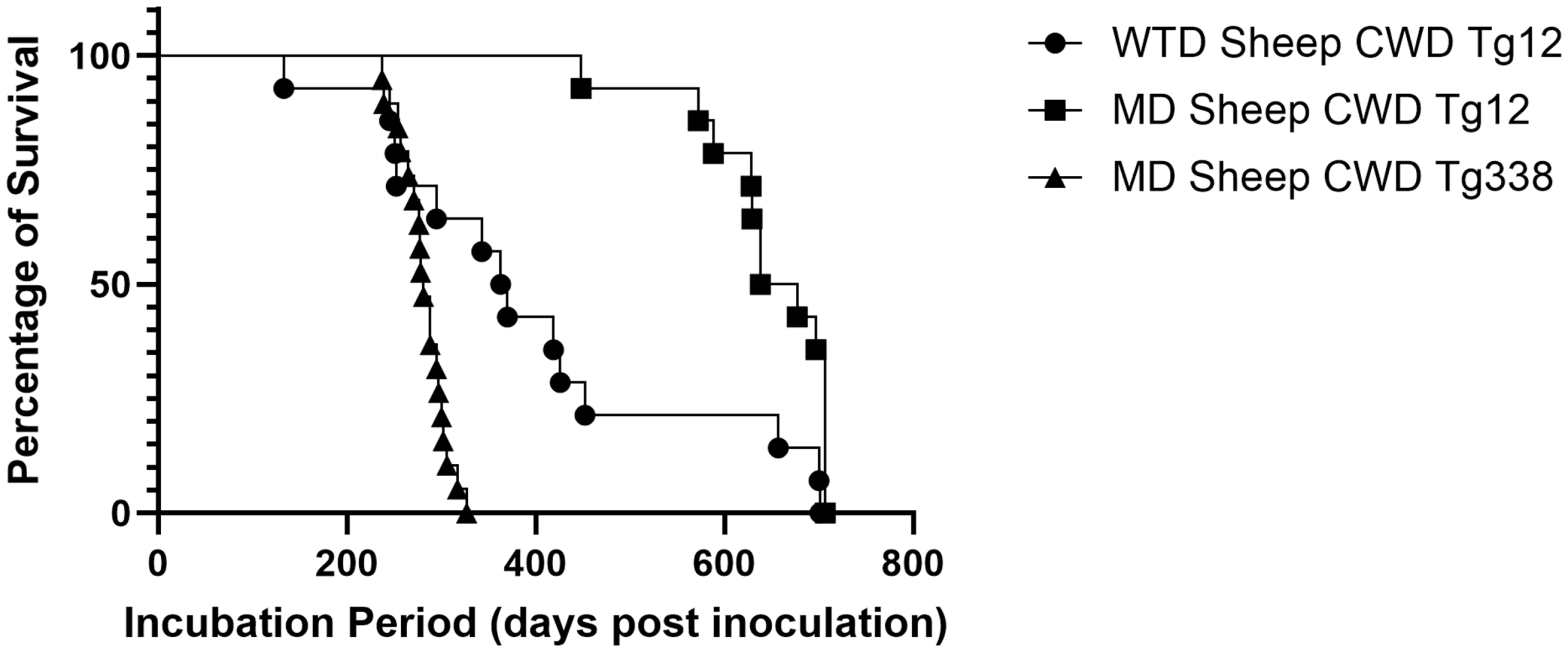
Incubation periods in transgenic mice. The graph above depicts incubation periods for cervidized (Tg12) and ovinized (Tg338) mice. Passage of the WTD sheep CWD agent into Tg12 mice (circle) resulted in a mean incubation period of 377 days. Passage of the MD sheep CWD agent resulted in a mean incubation period of 646 days in Tg12 mice (square) and 282 days in Tg338 mice (triangle).

Passage of the WTD sheep CWD agent into cervidized mice resulted in an attack rate of 83% with a mean incubation period of 377 days (Figure 5), while passage into ovinized mice resulted in 0% attack rate. These results were compared with the passage of the MD sheep CWD agent into cervidized and ovinized mice. There was positive detection in ovinized mice with a 100% attack rate and an incubation period of 282 days, whereas cervidized mice showed an 86% attack rate with an incubation period of 646 days (Figure 5).

### Cervidized mice western blots

**Figure 6:**
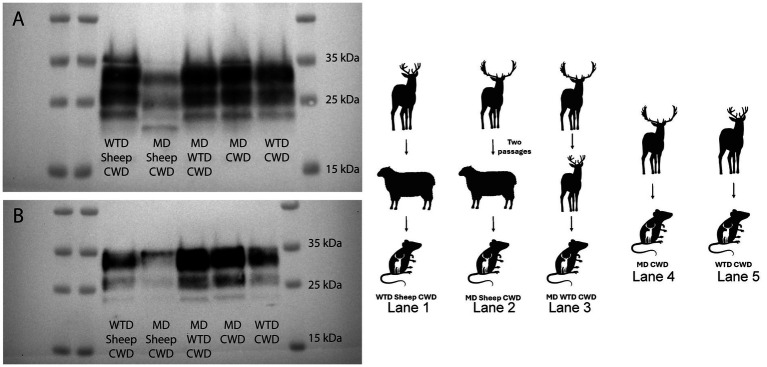
Cervidized mice western blots. Western blots were performed with monoclonal antibodies SHA31 **(A)** and 12B2 **(B)**, utilizing brain tissues of cervidized mice (Tg12) inoculated with the chronic wasting disease agent from various sources. The western blot performed with SHA31 **(A)** shows that the mule deer sheep chronic wasting disease agent has a different molecular profile with four bands compared to other lanes with only three bands. Samples probed with antibody 12B2 are difficult to differentiate in the western blot performed.

Cervidized mice were used to compare the WTD sheep CWD agent to the CWD agent from other cervids. Five groups of cervidized mice were inoculated with different prion agents. Group one was the WTD sheep CWD agent, group two was the MD sheep CWD agent, group three was the MD WTD CWD agent, group four was the MD CWD agent, and group five was the WTD CWD agent. The results showed that the MD sheep CWD agent (Figure 6, lane 2) displayed a different molecular profile with antibody SHA31 compared to the other groups. The MD sheep CWD agent appeared to have four bands, the unglycosylated band being lower than the other group’s unglycosylated band with antibody SHA31. To determine if there was a difference in PK cleavage point, N-terminal antibody 12B2 was used, and all five groups appeared to have similar molecular profiles (Figure 6).

### Ovinized mice western blot

**Figure 7:**
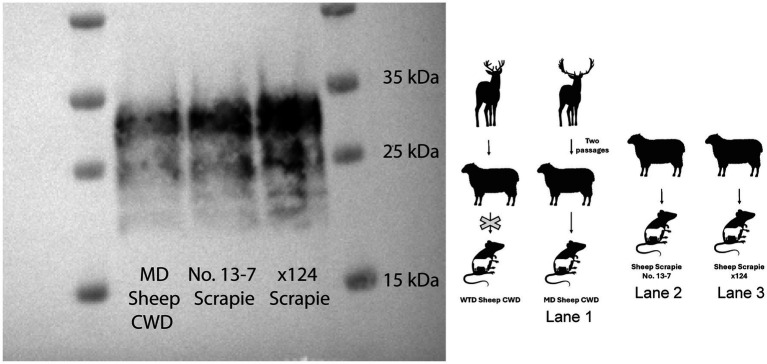
Ovinized mice western blot. The animal graphic aside the western blot displays the layout with the passage of white-tailed deer sheep chronic wasting disease agent into ovinized mice (western blot not performed as no infection occurred), the mule deer sheep chronic wasting disease agent into ovinized mice (lane 1), and two strains of classical scrapie, including No. 13-7 scrapie (lane 2) and x124 scrapie agent, into ovinized mice (lane 3). All samples on this western blot are ovinized mice (Tg338) probed with monoclonal antibody SHA31.

In order to determine the transmission potential of the WTD sheep CWD agent and the MD sheep CWD agent to sheep, ovinized mice were inoculated. The WTD sheep CWD agent did not transmit to ovinized mice; however, the MD sheep CWD agent did transmit to ovinized mice. A western blot was performed on the MD sheep CWD agent to compare the molecular profile to the scrapie agent in ovinized mice. The results showed that the MD sheep CWD agent had a molecular profile similar to two strains of classical scrapie, including No. 13-7 scrapie and x124 scrapie (Figure 7).

### Conformational stability assay

**Figure 8:**
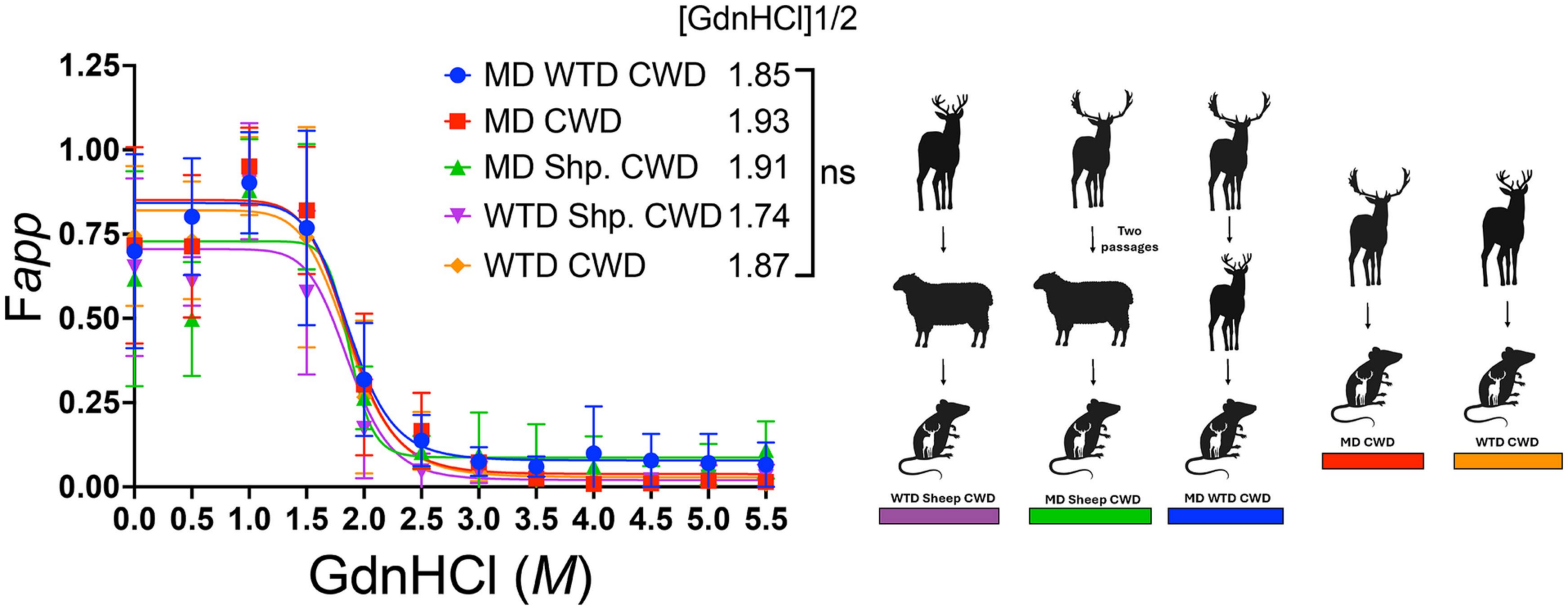
Conformational stability assay in cervidized mice. The conformational stability graph displays all brain samples from cervidized mice inoculated with the chronic wasting disease agent from various sources. The denaturation curve follows progressive amounts of guanidine hydrochloride (GdnHCl) on the x-axis while measuring the apparent fractional change of PrP^Sc^ at half-maximal amount of GdnHCl_1/2_ on the y-axis.

To investigate the possibility of differences in strain stability between the CWD agents from various cervids transmitted to sheep, conformational stability was evaluated on all cervidized mouse brains. Half-maximal denaturation of PrP^Sc^ with GdnHCl occurred in group one at 1.74 M, group two at 1.91 M, group three at 1.85 M, group four at 1.93 M, and group five at 1.87 M. Results showed no significant stability differences between groups, regardless of inoculum source (Figure 8).

## Discussion

### Donor source of the CWD agent affects transmission to sheep

The results demonstrated that sheep are susceptible to the CWD agent from WTD when intracranially inoculated, but only the CNS tissues show positive PrP^Sc^ accumulation, which is different from sheep intracranially inoculated with the agent of CWD from MD where there is PrP^Sc^ accumulation in both the brain and lymphoid tissues (15). Additionally, we have shown that this same WTD sheep CWD agent can be transmitted to cervidized mice, but not ovinized mice. In contrast, the MD sheep CWD agent positively transmits to cervidized and ovinized mice. When a western blot was performed on all of the cervidized mice with various CWD sources, the MD sheep CWD agent displayed a profile different from the other groups. When the monoclonal antibody SHA31 was utilized, the MD sheep CWD agent displayed four bands, different from the other cervidized mice, which displayed only three bands on the blot.

The differing transmission patterns of these two sheep CWD agents (WTD and MD) align with previous research (13–15), which indicates that sheep are more susceptible to the MD CWD agent than to the WTD CWD agent. One possibility for these agents to show different transmission results could be a difference in host *PRNP* genotypes. The MD CWD agent caused disease in sheep expressing the AVRQ genotype (15), while the WTD CWD agent affected sheep with the ARQ genotype (Table 1). These results suggest that the host genotype could play a critical role in the successful transmission of the CWD agent.

### Comparison to other research in sheep CWD transmission

Transgenic mice expressing the cervid and ovine prion protein genes are robust models for the transmission of both the CWD and scrapie agents. Previous studies have successfully demonstrated positive transmission of the CWD agent to cervidized mice and the sheep scrapie agent to ovinized mice (19). Notably, research comparing the transmission of the elk sheep CWD agent showed efficient transmission to both cervidized and ovinized mice (19), which highlights the potential for interspecies transmission of the CWD agent.

### Risk of CWD infection in sheep population varies depending on the original source of CWD

Currently, no known cases exist of sheep being naturally infected with the CWD agent. Interspecies transmission remains a slight possibility due to the geographic overlap between the CWD agent and sheep populations. These experimental studies suggest that the WTD CWD agent can be transmitted to sheep intracranially and then subsequently to cervidized mice. While intracranial inoculation is a valuable method for assessing disease progression and host susceptibility in experimental settings, results may not fully reflect natural transmission dynamics. Notably, the study reveals differences in transmission to ovinized mice when inoculated with the WTD sheep CWD agent compared to the MD sheep CWD agent. Overall, the data suggest that the WTD CWD agent is unlikely to pose a significant risk to sheep, but it could potentially be transmissible back to the cervid population. In contrast, the MD sheep CWD agent could pose a greater risk to both the cervid and sheep populations. This study is significant as it will benefit the cervid and sheep industry, as well as researchers in the field. Future work will focus on further characterizing the sheep CWD agent from WTD and MD when passed back into both large animal and mouse models.

## Data Availability

The original contributions presented in the study are included in the article/supplementary material, further inquiries can be directed to the corresponding author.
